# Structural and Psycho-Social Limits to Climate Change Adaptation in the Great Barrier Reef Region

**DOI:** 10.1371/journal.pone.0150575

**Published:** 2016-03-09

**Authors:** Louisa S. Evans, Christina C. Hicks, W. Neil Adger, Jon Barnett, Allison L. Perry, Pedro Fidelman, Renae Tobin

**Affiliations:** 1 Geography, College of Life and Environmental Sciences, University of Exeter, Exeter, United Kingdom; 2 Australian Research Council Centre of Excellence for Coral Reef Studies, James Cook University, Townsville, Australia; 3 Lancaster Environment Centre, Lancaster University, Lancaster, United Kingdom; 4 School of Geography, The University of Melbourne, Melbourne, Australia; 5 Oceana, C/Leganitos, 47, 28013, Madrid, Spain; 6 Sustainability Research Centre, University of the Sunshine Coast, Sunshine Coast, Australia; 7 Centre for Sustainable Tropical Fisheries and Aquaculture, School of Earth of Environmental Science, James Cook University, Townsville, Australia; University of Western Australia, AUSTRALIA

## Abstract

Adaptation, as a strategy to respond to climate change, has limits: there are conditions under which adaptation strategies fail to alleviate impacts from climate change. Research has primarily focused on identifying absolute bio-physical limits. This paper contributes empirical insight to an emerging literature on the social limits to adaptation. Such limits arise from the ways in which societies perceive, experience and respond to climate change. Using qualitative data from multi-stakeholder workshops and key-informant interviews with representatives of the fisheries and tourism sectors of the Great Barrier Reef region, we identify psycho-social and structural limits associated with key adaptation strategies, and examine how these are perceived as more or less absolute across levels of organisation. We find that actors experience social limits to adaptation when: i) the effort of pursuing a strategy exceeds the benefits of desired adaptation outcomes; ii) the particular strategy does not address the actual source of vulnerability, and; iii) the benefits derived from adaptation are undermined by external factors. We also find that social limits are not necessarily more absolute at higher levels of organisation: respondents perceived considerable opportunities to address some psycho-social limits at the national-international interface, while they considered some social limits at the local and regional levels to be effectively absolute.

## Introduction

Research is beginning to challenge the assumption that adaptation can avert all impacts from climate change [1–4]. Under certain conditions adaptation fails [3,5,6]. It is, therefore, necessary to understand what adaptation can and cannot do, and to identify limits to adaptation–the points at which it fails to alleviate impacts or creates more vulnerabilities than it addresses [7,8]. Recognising limits helps to prioritise adaptation responses, avoid strategies that may fail, and identify places and sectors that require compensation to offset probable losses.

Studies of limits to climate change adaptation have focused on ecological, technical and economic limits. Limits to adaptation include for example: the temperature at which a species fails to acclimatise physiologically to warmer temperatures [9,10]; the amount of sea level rise that technologies like a seawall can no longer hold back, or; the point at which adaptation measures cost more than the impacts they avert [11]. Different adaptation strategies such as adapting to warmer ocean temperatures through acclimatisation, evolutionary adaptation or distributional range shifts are subject to distinct limits [10]. Limits thereby remain highly uncertain [12] but are typically viewed as being insurmountable or absolute [7,8,13].

Emerging evidence in the social sciences suggests that social factors, related to how people understand and experience climate change, can also manifest as limits to adaptation [7,8]. Importantly, these social limits can become apparent before other limits are reached, for instance, specific species adaptations or engineering solutions may be viewed as invasive or undesirable and so prevented from occurring. Nevertheless, there has been relatively little empirical research investigating the nature and mutability of social limits to adaptation.

We investigate social limits to human adaptation, focusing on climate-sensitive industries in the Great Barrier Reef region, Australia, where climate change impacts are already experienced and projected to be significant in future [9]. The region refers to two broad administrative units: the Great Barrier Reef Marine Protected Area (345,000 km^2^) and an area encompassing the adjacent coastline and river catchments (425,000 km^2^). We identify social limits to specific adaptation strategies in the fishing and reef-based tourism sectors, distinguishing factors operating within and between reef-industries (micro-level), at the regional level between reef industries and other land-based sectors (meso-level), and at the national-international level between reef industries and the global economy (macro-level). We argue that social limits are significant but not necessarily absolute. We hypothesise that they are more mutable at lower levels of organisation where it is potentially feasible, through concerted action by those impacted by and adapting to climate change, to manage influencing factors to some degree to move, delay or overcome social limits.

## Conceptualising Limits to Adaptation

Barriers and limits to adaptation are conceptually distinct though definitions vary. Where barriers block or divert the adaptation process, limits often represent more absolute thresholds [13,14]. Dow et al. [14] suggest, for example, that social limits to adaptation are reached when people experience an unacceptable loss of yield above the temperature threshold at which ecological adaptation in rice pollination can occur. In the example, social limits arise from experiences of ecological limits, and once a limit is reached people must transform their behaviour to avoid risk [14,15].

In this paper we argue that social limits can apply to *specific* adaptation strategies, can arise prior to thresholds for ecological or technical limits, that they are more or less mutable for different sets of actors across scales and that once reached other adaptation options, if available, may be considered before transformation is needed [16]. We define limits as the point at which adaptation is not just *inefficient* (as influenced by barriers) but *ineffective at reducing the vulnerability of those undertaking adaptation*. In line with Felgenhauer [17], once limits are reached the existing adaptation strategy may still reduce impacts but not sufficiently to prevent unacceptable losses. We suggest that any particular factor can act as a barrier or manifest as a limit depending on when and how it influences adaptation action and outcomes [18]. For example, uncertainty can both prevent adaptation and reduce the benefits received by dampening adaptive action. Barriers reduce *added benefit*. However, where people adapt in a context of uncertainty and make more risky decisions that mean adaptation losses are greater than benefits, uncertainty is a limit to adaptation.

There is increasing recognition that social barriers and limits exist along a continuum [8,13,18]. The IPCC fifth assessment report on adaptation opportunities, constraints and limits states that: “Various constraints [barriers] … can, if sufficiently severe, pose limits to the ability of actors to adapt to climate change … Because adaptation limits relate to adaptation resources and attitudes to risk that may change over time, some limits may be viewed as “soft” or time sensitive” ([8]: 919). Here we seek to understand when particular factors effectively become limits to adaptation as an important empirical and policy question.

Social limits, broadly defined to distinguish from biological and technical limits, arise through psycho-social processes related to what we perceive, know and value [19–23]. They can also stem from organisational and institutional structures such as policy-making and markets [24–26]. To what extent social actors have control over and can significantly influence these psycho-social and structural factors determines how mutable or effectively absolute limits are for different people and industries. We explore the perceived mutability of limits as they emerge across scales.

## Methods

We used a design based on a single multi-scale system and qualitative methods [27] to explore *perceived social limits* to human adaptation in reef-based industries vulnerable to climate change. Data came from expert elicitation of respondents purposively selected to represent the primary stakeholders engaged in use and management of the Great Barrier Reef. To launch the project, its aims and activities were discussed with the Great Barrier Reef Marine Park Authority and circulated on national and regional information networks (e.g., NCCARF’s adaptation network). To recruit participants for the workshop, we emailed an invitation to selected representatives of twenty-four organisations that manage or represent the fisheries and tourism industries in the Great Barrier Reef. We followed up where necessary with telephone calls. Representatives from sixteen key organisations participated in this research (see **S1 Table**). Experts included representatives of state and local governments, the recreational fishing, commercial fishing and tourism industries, non-governmental organisations and scientific organisations. As representatives of key organisations or networks the experts provided collective or organisational rather than personal perspectives. All participants partook voluntarily and provided written informed consent. Consent was obtained at the start of each workshop and interview. Full anonymity was not possible given that data were gathered in workshop settings, however, participants were assured that quotations would not be directly attributed to individuals or their organisations.

To understand to what extent social limits were shared or unique to particular sectors and to encourage diverse discussion over the mutability of limits we selected to collect data through multi-stakeholder workshops. The lead author facilitated two workshops in March and April 2012 in two different locations along the Queensland coast (Townsville and Cairns) (n = 9 and n = 11, each workshop included at least one representative of industry, government, the non-governmental sector and science and was 5–6 hours duration). The workshops followed an open-ended, exploratory format guided by broad research questions (below). Ground rules were established with participants to ensure wide participation and avoid conflict, and two other members of the research team helped guide discussion and document key points [28]. Semi-structured key-informant interviews with respondents who could not attend the workshops were also conducted between March and June 2012 at a location chosen by the respondent (n = 6, 1–2 hours duration). These interviews aimed to supplement the workshops to ensure the relevant organisations were represented and sufficient views from industry were captured. Interviewees included representatives of the commercial fishing, recreational fishing and tourism industries. Data saturation around experiences of industry adaptation suggested that a sufficient sample of organisational perspectives was included in the research [29].

The research questions explored: i) desirable adaptation goals; ii) adaptation strategies and actions available to industry, and their potential to deliver desirable outcomes, and; iii) factors preventing particular strategies and actions from being pursued, or *from achieving desired goals (social limits)*, *and to what extent these could be addressed by concerted effort (mutable)*. Respondents considered interactions or influences across organisational levels: within and between individual sectors (micro); within the region, accounting for non-reef activities and industries (meso), and; within the national to international sphere (macro). The multi-stakeholder workshop approach (and supplementary interviews) was used to develop consensual views on limits rather than to understand differences in perspective among organisations. Respondents identified limits that were specific to sectors but these did not arise through disagreement in the data or data disaggregation, rather through the collective understanding of a range of stakeholders with intricate knowledge of these sectors.

Data were digitally recorded, professionally transcribed and analysed using QSR NVivo v9. The analysis used a list of codes to identify desired adaptation outcomes; specific adaptation strategies and actions mentioned; factors referred to as limiting, blocking, constraining, or hindering adaptation; the organisational levels at which limits are perpetuated, and; statements about opportunities or solutions. Note, respondents did not intuitively differentiate barriers and limits to adaptation (see also Coultard [30] on the continuum of short-term coping and long-term adaptation). To avoid limiting topics and discussion, respondents were able to use a range of descriptors in the workshops and interviews. During data analysis, to isolate limits, the research team conducted a second level of coding to identify factors linked specifically to adaptation failing to deliver desired outcomes. We then also coded factors as primarily psycho-social or structural. Finally, where respondents referred to issues as opportunities rather than or as well as constraints, or where respondents provided solutions to adaptation failures we consider these to reflect perceptions of higher mutability of associated limits. Findings were sense-checked with key stakeholder representatives in a dissemination workshop in August 2011 (n = 7).

## Results

### Adaptation strategies

Respondents discussed a range of adaptation strategies and examples of specific adaptation actions that had been trialled or considered within fisheries, tourism and other sectors (Table 1). They argued that adaptation was an extension of resource management and effective entrepreneurship and was not only relevant to climate change. As such, improved business planning and stewardship (going beyond legislated resource management) were identified as important strategies (see [31] for more detail on these findings).

**Table 1:** Examples of adaptation strategies and actions in the Great Barrier Reef fishing and tourism industries.

Adaptation strategies	Examples of adaptation actions
**Business planning:** Any combination of improved forecasting, financial management, marketing and networking.	Increased resolution climate forecasting for primary industries.
	Improved monitoring and forecasting of bleaching—BleachWatch.
	Computer packages for modelling business scenarios in tourism and fisheries.
	Networking of Climate Champions in farming communities across Australia
**Diversification:** Can occur through more varied gears, catches, or sources of income in the fishing sector and through more diverse services, locations or income sources in the tourism sector.	Diversification into high value seafood commodities.
	Temporary diversification of product in the commercial reef line fishery after Cyclone Hamish.
	Targeting more inter and intra-state tourism.
	Development of a “Great Eight” concept encouraging tourists to value interacting with identified iconic species, mirroring the “Big Five” in wildlife tourism (Buffalo, Elephant, Leopard, Lion, Rhinoceros).
	Tourist trips on commercial trawlers.
	Diversified activities undertaken by recreational fishers e.g., snorkelling and picnicking.
**Effort management:** May involve increased effort to counteract impacts, or reduced effort to minimise costs and avoid exacerbating impacts. May be temporary or permanent.	Incremental increase in effort e.g., through improved technologies.
	Caps on numbers of tourism operators in sensitive locations. E.g., Lord Howe Island, Hinchinbrook.
	Prohibit harvesting of 16 species of herbivorous fish in the aquarium fishery, following bleaching events.
	Effort buy-outs in commercial fisheries.
**Mobility & migration:** May involve changes in the spatial distribution of operators and industry sectors through permanent migration or temporary shifts in response to climate change impacts	Recreational fishers travelling to the Northern Territory.
	Commercial fishers shifting fishing grounds following Cyclone Hamish.
	Commercial fishers buying licenses in other states.
	Change from spatial to stock entitlements in the aquarium fishery.
	Flexible permitting of tourism operations following flood / weather events.
**Stewardship:** Underpinned by self-organised ability to manage ecosystems, demonstrated by industries dependent on the natural environment (with implications for the degree of external regulation).	Impact assessment, auditing, and certification e.g., eco-labelling and carbon footprint assessment.
	Accreditation and certification programmes linked to extended licensing through Eco Tourism Australia.
	Data and statistics on the ‘State of the Environment’.
	Increased public communication e.g., worksheets to tourists, TV commercials to support Reef Guardian Fishers.

Respondents also identified and discussed desirable adaptation goals, which we have classified as ecological sustainability, economic viability, and enjoyment. Representatives of commercial fishing and tourism stated that these sectors were concerned with maintaining (or improving) both sustainability of the reef and the profitability of their sector, with some operators also prioritising enjoyment and the maintenance of the lifestyle values of commercial fishing. Recreational fishing representatives suggest this sector focused primarily on enjoyment of the reef, linked to access and ecological sustainability.

Respondents debated to what extent particular adaptation strategies could deliver these desired adaptation goals now and in the future. Our analysis showed that different adaptation strategies are available to each sector (Table 2). For example, commercial fishers can employ a broad suite of adaptation strategies to achieve their three goals. Diversification, for instance, is primarily employed to maintain or improve the economic viability of commercial fishing businesses in times of uncertainty. Other strategies, like improved business planning, can purportedly achieve multiple adaptation outcomes. Note, this analysis is at a sectoral level and does not suggest that every individual operator has the capacity to employ the full suite of strategies. In contrast, fewer adaptation strategies are employed by or available to the recreational fishing sector.

**Table 2:** Adaptation strategies and desired adaptation outcomes in the Great Barrier Reef fishing and tourism industries.

Strategy	Outcome		
	*Economic viability*	*Ecological Sustainability*	*Enjoyment*
Business planning	CF, RT	CF, RT	CF
Diversification	CF, RT		
Effort management	CF	CF	CF
Mobility and migration	CF	CF	RF
Stewardship	CF, RT	CF, RF, RT	CF, RF

CF = Commercial fishing, RF = Recreational fishing, RT = Reef-based tourism

Through detailed multi-stakeholder discussion of the strategies potentially employed by different sectors and their ability to deliver desired outcomes respondents identified *perceived limits* to success. Here, our analysis revealed that distinct and multiple limits apply to the different sectors even within a single adaptation strategy, as detailed below.

### Social limits to adaptation strategies

Respondents identified both psycho-social and structural limits to particular strategies (Table 3) at each organisational level. Our data show that specific factors such as identity, market dynamics and co-ordinating institutions can act as limits across a range of adaptation strategies. As we demonstrate below, however, different sectors experience these factors as limits to adaptation in different ways. Importantly, we find that social limits are comprised of a composite set of interacting factors that drive and mediate how limits are experienced. For example, interactions between psycho-social and structural factors can enhance the extent to which a lack of collective action manifests as a limit to adaptation by particular actors. We considered these interactions important and hence, going forward, refer to the psycho-social, structural *and* combined interactions (collective action) as limits where their combination causes adaptation to fail to deliver desired outcomes. Below we outline selected examples of how psycho-social and structural factors interact to create limits to adaptation for different sectors across levels of organisation. The examples reflect the main areas of discussion in the workshops and interviews at each organisational level rather than explaining the full list of limits included in Table 3.

**Table 3:** Key social limits associated with adaptation strategies in the Great Barrier Reef fishing and tourism industries.

Adaptation strategy	Factors that manifest as psycho-social limits	Factors that manifest as structural limits
Business planning	Self-perception	Property rights
	Identity	Political voice
	Reputation	Market variability
		Financial externalities
Diversification	Reputation	Market demand
	Knowledge	Skills
Effort management	Self-perception	Co-ordinating institutions
	Perception of others	Market competition
Mobility & migration	Family ties	Market networks
		Market demand
Stewardship	Self-perception	Co-ordinating institutions
	Identity	Property rights
	Reputation	Environmental externalities

#### Micro level limits

Respondents argued that factors underpinning a lack of collective action within and between reef industries acted as important social limits to adaptation. Desired adaptation outcomes were often not achieved through individual action, only through a ‘whole-of-industry’ or integrated approach (see also [32]). Factors undermining collective action and thereby business planning, effort management, and stewardship included different perceptions of risks and responsibilities between sectors, a lack of effective industry representation, and the absence of institutions to co-ordinate collective action within and across sectors (Table 3).

In the commercial Coral Reef Fin Fish Fishery, respondents viewed the failure to mediate resource allocation within and between this sector and the recreational fishery as a considerable adaptation challenge. According to a representative of this fishery, following Cyclone Yasi (2011) commercial fishers volunteered to shelve ~23 percent of their quota for 12 months to allow coral trout stocks to recover. However, fishers expected that fisheries managers would not put sufficient controls in place to ensure that recreational fishers, who also exploit this fishery, would not access and extract the fish stocks safe-guarded by adaptation in the commercial fisheries sector. Individual recreational fishers often do not perceive the aggregate impact of their activities on the reef (see Table 4 for illustrative quotes). The sector is highly dispersed with relatively low levels of representation making flexible regulation of the sector difficult. As a result, respondents argued that commercial fishers pursuing a strategy of voluntarily reducing quota would fail to achieve stock recovery and, by extension, sustainability and economic viability of the Coral Reef Fin Fish Fishery following the extreme weather event. Hence, interactions between recreational fishers’ self-perceptions and lack of self-regulation, and the lack of institutional co-ordination mechanisms manifest as a limit to adaptation for commercial fishers. These factors would render adaptation strategies *ineffective* rather than *inefficient* considering the expected impacts of recreational fishing on the 23 percent of quota preserved.

**Table 4:** Interview data to reflect the range of factors that can limit adaptation to climate change across sociological and organisational scales.

	Psycho-social	Structural
**Micro-level:** Industry-level collective action	**Perceptions of risk and responsibility**	**Co-ordination and regulation**
	*…For every one commercial fisherman we‘ve probably got about a thousand recreational fishermen*. *They don’t see how big that impact can be*. Recreational fishing representative	*If they [fishery managers] don’t do anything they can’t get in trouble*.* *.* *.*We have a 10 year plan for our fishery*, *we’re 7 years in*, *the recreational catch has gone up*, *we reckon*, *by about 280 tonne of trout a year and the Management won’t break into the plan and change it*. Commercial fishing representative
	*Because [recreational fishing] don’t have those organisational units*, *it just makes it much more difficult for that sector to become involved…* Commercial fishing representative	*We have an accreditation program that’s been agreed to…You can get a 14 or 15 year permit if you’re GBRMPA and Eco Tourism accredited*. *You can get a seven or 10 year permit if you’re not*. Tourism industry representative
**Meso-level:** Multi-sectoral collective action	**Perceptions of risk, responsibility and identity**	**Economic development, private property rights and externalities**
	*Australia can’t feed itself*, *it’s mesmerised with coal*. *You can’t eat coal*, *it’s not a renewable resource but seafood is—it comes from pristine waters*, *it’s managed in a sustainable manner*, *we’ve been ticked off with all the hoops we’ve jumped through…*. *But you can’t help but think they don’t want us*. *They’re getting rid of us*. *It’s death by a thousand cuts and they’re wearing us down*. Commercial fishing representative	*In Abbott Bay they’re putting in a new coal terminal*. *They’re [government] saying ‘well we’re not going to stop the development’*. *Certainly in Queensland*, *it’s about identifying those people that are going to lose out and compensating them*. *But to me*, *the environment is definitely going to lose in the long term*. *How’s that going to affect our whole industry*? *It is a big worry*. Commercial fishing representative
		*Coastal development is probably one of the biggest threats that we have*. *We tried with the coastal management plan a number of years ago but it’s just gone from bad to worse*. *It’s not really getting the political mileage that it needs*. Government representative
**Macro-level:** Multi-national arrangements	**Reputation**	**Market demand**
	*We’re probably catching fish from the most sustainably managed reef ecosystem in the world*. *Now how good a job are we doing at marketing that*? *Pretty awful*. Commercial fishing representative	*When cyclone Hamish came along 80% of the [Reef Line] industry moved but they couldn’t move too far from Bowen because that’s where they got the best price for their product*. *So*, *they all moved on top of each other*. *Their mobility was limited not by any regulation*, *not by any limit of fish distribution*, *but by where there market was*. Representative of the scientific community
	*We want to stir the political debate in a sense—the conservation movement does—but it is very difficult to get a differentiated message across to say ‘there’re all these threats to the reef*, *but wait a minute*, *it’s actually very well managed and probably in better nick than any other reef in the world’*. *That is a big conundrum for us*, *to get that subtle message across in the media*. Tourism industry representative	

By contrast, respondents credited wide representation and effective co-ordination of tourism operators by industry organisations with facilitating effective adaptation through business planning and stewardship. Respondents detailed how industry organisations enabled tourism operators within (different reef-based operators) and across sub-sectors (alpine to reef sectors) to adapt to seasonal requirements and extreme weather events by sharing staff, thereby minimising business costs in quiet periods while retaining skilled labour. Respondents also described how stewardship and best-practice strategies are promoted through industry accreditation schemes linked to extended permits. Attaining Eco Tourism Australia accreditation, a scheme approved by the Great Barrier Reef Marine Park Authority, can enable operators to obtain a five to seven year extension on operator permits (Table 4).

Note, like uncertainty, the factors underpinning a lack of co-operation and co-ordination, can be either barriers or limits to adaptation. They act as barriers where actors perceive unfair outcomes from stewardship practices but would nevertheless receive benefits. In the example given, they are identified as limits because respondents argued that potential *benefits from stewardship would be negated by the lack of mechanisms to mediate competition for resources*. Importantly, these limits apply to specific fisheries with issues of resource availability, like the coral trout fishery, but would not necessarily manifest as limits in other fisheries in the same way.

#### Meso level limits

Respondents argued that meso-level factors underpinning a lack of collective action between reef and non-reef sectors within the Great Barrier Reef region were also experienced as limits to adaptation. Effective adaptation by reef industries depends on maintenance of important public goods (e.g., water quality and community safety), which are impacted by land-based sectors beyond the sphere of influence of reef industries. Negative externalities that emerge from the predominance of terrestrial private property rights, and the powerful interests and deeply embedded identities linked to Australian agricultural industries (e.g., cattle grazing and sugar cane production), mining, and coastal lifestyles (e.g., beach-front properties) represent a limit to business planning and stewardship by reef industries (Tables 3 and 4).

In the Great Barrier Reef region, institutions and incentives that protect and reinforce private rights on land represent a particularly intractable set of limits to adaptation for the reef and its industries [33]. The declining water quality in the Great Barrier Reef resulting from siltation and pollution run-off from current land-use practices in the catchment is already a high management priority [34]. Respondents argued that as graziers and farmers become more climate-stressed and undertake their own adaptations on private land, the externalities impacting marine ecosystems and industries are likely to increase. Beyond a certain threshold these externalities will undermine any efforts by reef operators and managers to improve outcomes through adaptation.

Respondents suggested that individual property rights on land exercised in coastal development decisions would also produce externalities that could limit adaptation of reef industries in two ways. First, coastal property owners have the right to fortify their properties against sea-level rise. If many individuals do so, the implications for marine ecosystems and dependent sectors are considerable: fortification presents a limit to ecological adaptation preventing distributional shifts of important coastal habitats, including mangroves, and impacts on ecosystem health and fisheries productivity [35]. Again, respondents suggested that beyond a certain point these activities would render their efforts to adapt through stewardship activities ineffective. Second, the current building and safety standards that coastal residents are encouraged to adhere to are deemed inadequate by respondents in the face of more intense extreme events, and there are no incentives for coastal residents to exceed these standards. Respondents argued that the high costs of resultant emergency response are then passed on to coastal fisheries and tourism businesses as financial externalities (insurance premiums and recovery levies). Limits to adaptation by individual businesses that build or retrofit infrastructure to high standards are experienced when other individuals do not act collectively in a way that improves community safety, reduces the cost of emergency response and subsequently prevents the transfer of significant financial externalities (see also [36]). In aggregate, the individual actions of land-users produce externalities such as competition for space, siltation and pollution [34], and emergency response costs that, beyond a certain point, render ineffective (rather than inefficient) key adaptation strategies by reef industries.

Participants noted the strong identities tied up with coastal lifestyles, mining and agricultural industries, which drive political interests and are used to justify the actions and rights of land-based industries in the Great Barrier Reef region thereby reinforcing structural limits to adaptation in reef-based industries. Federal and state governments are perceived to be unwilling to act against the economic interests of their more powerful constituents, thereby prioritising the interests of influential (but not necessarily majority) groups over the long-term sustainability of the reef (see also [37]), and marginalising groups like commercial fishers who reported feeling ‘unwanted’ and undervalued (Table 4).

#### Macro level limits

Reef industry representatives further confirmed that adaptation is highly sensitive to macro-level factors that reflect the interplay between national and international policy. These factors appear to primarily influence the economic viability of reef industries. Structural characteristics of the international marketplace that drive market demand and competition present a limit to adaptation through effort management, and mobility and migration (Table 3). The national and international reputation of reef industries was also identified as an important limit to adaptation strategies such as business planning, diversification and stewardship.

Commercial fisher representatives argued that many adaptation strategies will not deliver desired adaptation outcomes like improved economic viability because they do not directly address the factors that constrain profitability. In the East Coast Trawl Fishery buy-back of effort to permanently remove capacity and free up fishery resources for those who remain in the sector was under consideration. However, industry representatives posited that profitability is not constrained by resource availability but by trade liberalisation and international market competition. Fishers in the Great Barrier Reef have moved from being ‘price-makers’ to ‘price-takers.’ In this context, adaptation through effort management will fail to increase profits and improve economic viability and enjoyment for those remaining in the industry. In short, it is not the abundance of prawn, for example, nor access to the stock that constrain the profitability of the trawl sector but the price fishers get for their catch, which in turn is under pressure from cheaper seafood imports. Reducing sector-level effort would not reduce vulnerability for remaining operators in this context. In contrast, representatives suggest that permanent effort reduction may be an effective adaptation strategy in the Coral Reef Fin Fish fishery where resource allocation (i.e., the abundance of the resources and its distribution among the commercial operators and between commercial and recreational fishers) is a concern.

Market characteristics represent a different limit to adaptation in the Coral Reef Fin Fish fishery. This fishery primarily targets coral trout (*Plectropomus spp*) for the valuable live reef fish food trade to Asia. The Chinese market, in particular, values the red *Plectropomus leopardus*, which is more abundant in the southern reef [38,39]. Coral trout are sensitive to extreme weather events and commercial catch rates purportedly declined following both Cyclone Hamish (2009) and Cyclone Yasi (2011). Following the former event, fishers attempted to migrate away from heavily impacted southern reefs. However, operators congregated around key ports that are known to pay higher prices, such as Bowen in the central Great Barrier Reef, thereby concentrating fishing effort in particular areas and exasperating disturbance to the fish stock in these sites [40]. Tobin et al. [40] report that following these experiences a few fishers exited the fishery. This demonstrates that a limit to adaptation, like market competition, can differentially affect sectors as well as various operators within a sector.

Market forces are also a limit to climate change adaptation in the reef-based tourism sector. According to respondents, a strong Australian dollar, high energy and labour costs, and stricter regulatory frameworks relative to emerging tourism industries in East Asia and the Pacific jeopardises the international competitiveness of the Great Barrier Reef’s tourism sector. This high competition reduces the benefits that can be gained from adaptation through improved business planning and product diversification that aim to maintain profitability in times of change. For some businesses under certain climate change conditions these factors can mean that impacts exceed benefits and limits to adaptation are reached.

Respondents argued that poor or declining industry reputations exacerbate the structural effects of market competition representing further limits to adaptation. Despite considerable progress in the stewardship of the Reef’s commercial fisheries, respondents highlighted a deeply held public perception of fishers as highly exploitative. This reputation reflects a limit to the potential for economic gains from business planning, diversification and stewardship, as benefits depend on achieving increased market share (Table 4). Tourism industry representatives also explained that while Queensland’s reef-based tourism services are aspirational and of high quality, consumer perceptions are pervaded by increasingly pessimistic views of the reef and its future prospects. Respondents suggested that information campaigns that have used the iconic Great Barrier Reef to highlight the importance of reefs and their vulnerability to global threats, in particular climate change, have created a perception abroad that the Great Barrier Reef is already highly degraded and not worth visiting. This compromises market share and profitability, presenting a limit to the potential for adaptation through business planning and diversification. At the point where climate change impacts exceed any benefits gained from market share changes, negative reputations represent important psycho-social limits.

**Fig 1:**
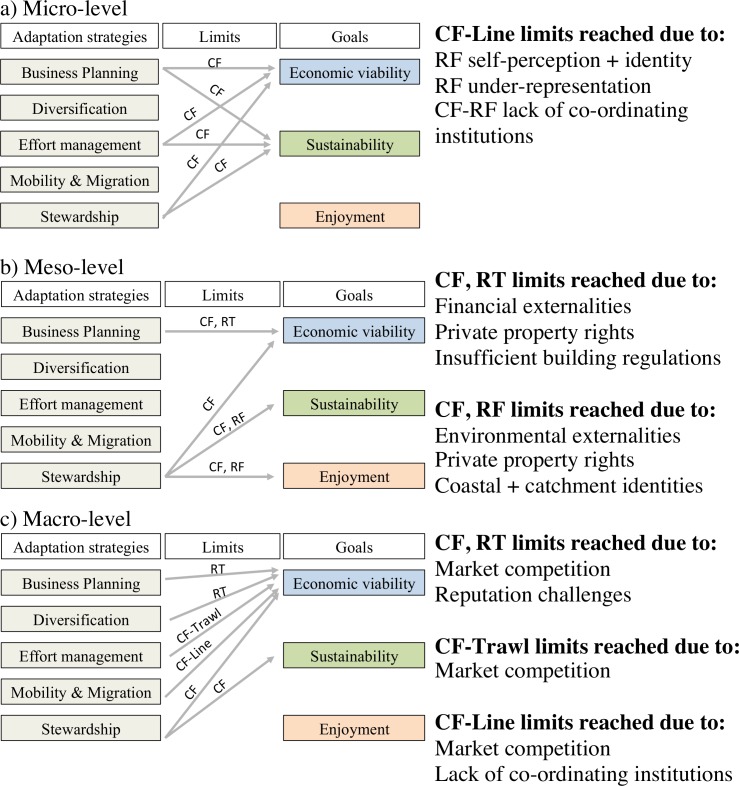
Summary of factors creating limits to adaptation across organisational levels in the Great Barrier Reef fishing and tourism industries. CF = Commercial fishing, RF = Recreational fishing, RT = Reef-based tourism.

The results presented above are illustrative of how psycho-social and structural factors can be experienced as limits to adaptation in different ways for different sectors across organisational levels (see Fig 1). The findings suggest that limits are highly context specific. For instance, effort management could effectively address vulnerabilities in the Coral Reef Fin Fish fishery but not necessarily in the East Coast Trawl fishery given the predominant influence of market competition in the trawl sector. The context specificity of social limits is an important insight for the management of limits to adaptation. Our research also uncovered some more generalisable findings about social limits to adaptation: first, that individual factors, such as identity, can play an important role across multiple sectors and organisational levels even where outcomes are distinct to particular adaptation actors, and; second, that social limits arise through different mechanisms or pathways particularly when understood across scales. These insights are elaborated on in the Discussion.

### Mutability of social limits to adaptation

At first glance it appears more conceivable to create incentives to motivate collective action within and between reef industries than to manage trade-offs in interests between reef and non-reef industries or regional and global actors. However, analysis of respondents’ perspectives on opportunities to overcome limits suggests that limits at higher levels of organisation are not necessarily the most insurmountable.

Our data suggest that certain limits at sectoral and regional levels, such as a lack of collective action underpinned by the self-perceptions of recreational fishers or the negative externalities driven by the strong identities of agricultural industries are more difficult to address than limits at the national-international level related to industry reputation. In particular, seemingly intractable limits emerge at the regional level where identities and values linked to land-use practices are defended through political influence and associated policy. Many land-based sectors appeal to coastal and agricultural identities to convey private property and individualism as fundamental, unalienable rights. Consequently, reef industry representatives argued that the ever-increasing coastal population and multiple uses of the Great Barrier Reef catchment considerably reduce the likelihood that externalities from land-use practices and associated limits to adaptation for reef industries will be significantly reduced or removed. For reef-based industries these social limits to adaptation are effectively absolute.

In contrast, in both the tourism and commercial fishing industries respondents see considerable opportunity to overcome psycho-social limits to adaptation at the national-international level by influencing consumer preferences and broad public perceptions. The tourism industry launched a campaign to promote the Great Barrier Reef as ‘The Best Managed Reef in the World’ to counter what they perceived as its declining reputation. Further, the federal and state governments provided an AU$12 million recovery fund for Queensland following Cyclone Yasi in part to counter perceptions that the region was ‘not worth visiting’ [41]. In fisheries, the Reef Guardian programme, a collaboration among federal and state agencies and fishing enterprises to encourage stakeholder stewardship, advertise Reef Guardian Fishers on national television to address misconceptions about the commercial fishing industry. Developing buy-local, green, and healthy food campaigns for commercial fisheries was widely viewed as a critical opportunity to address strong market competition from imported products, enhance public perceptions, and improve outcomes from adaptation across commercial fishing sectors.

## Discussion and Conclusion

Our empirical investigation advances the conceptual understanding of social limits to human adaptation. Social factors can act as either barriers or limits to adaptation depending on when and how they influence action and outcomes. Our study highlights many examples of where key, interacting psycho-social and structural factors might render adaptation *ineffective* rather than simply *inefficient* as a response to climate change. It also highlights how social limits can apply to specific adaptation strategies, be reached before ecological and technological limits, and how a single factor or set of factors may be experienced as a limit in some contexts but not others.

Our data highlights both psycho-social and structural dimensions to limits, which are closely linked in many cases. A large body of research, as synthesised in the IPCC fifth assessment report [8], has recognised the potential of psycho-social and structural factors to block or divert adaptation. Psycho-social and structural constraints (barriers) on adaptation include perceptions of risk, identity, lifestyle choices, competing values, property rights and rigid tenure arrangements, and a lack of co-ordinating institutions and appropriate regulatory structures [8]. Our research argues that under certain conditions these factors already do or are anticipated to limit adaptation in practice. Emerging research on limits supports our claim.

In their conceptual review Adger et al. ([16]: 338) ague that “the existence of diverse, and sometimes incommensurable, values held by the actors involved in decision-making around adaptation can act as limits if these values are not deliberated”. Many of the limits identified by respondents in our study relate to the conflicting interests and values of different actors playing out at multiple levels, including commercial and recreational fisheries, reef and non-reef industries, and local and global enterprises. In emerging empirical work, identities, sense of place, and conflicting values and interests are recurrent limits to adaptation. Identities and sense of place tend to arise at the micro-level as ‘self-imposed’ limits to adaptation, which are nevertheless extremely difficult if not impossible to shift [4,42]. For instance, Warner et al. [42] found that farmers in Costa Rica identified so strongly with being rice farmers that few adaptation strategies were available to effectively reduce their vulnerability to water stress and climate change. Conflicting values and interests emerge at multiple organisational levels: among single actor groups and, often, between different actors, industries and communities. Conflicting demands for water, for instance, were found to impose widespread limits to adaptation across Alpine, Riverine and Catchment ecosystems, industries and communities in Australia [18].

Other limits emerged from institutional inertia or mis-matches. This is illustrated in different ways by limits to stewardship in the Coral Reef Finfish Fishery (no control over recreational fisher access to the fishery) and limits to effort reduction in the East Coast Trawl Fishery (no control over market price). Institutional mismatches also played a role in perpetuating negative externalities from the adjacent coastline and catchment. Examples of structural limits to adaptation in the literature include the rigid tenure arrangements of ‘terroir’ which prevent relocation of wine producers in line with ecological adaptations of grape varieties [43], institutional inertia in planning policy and legislation [44] and, the failure of institutions or market mechanisms (e.g., water trading) to mediate conflicting water demands, which for downstream users can be experienced as significant limits to adaptation [18]. Relatively few studies appear to have examined limits to adaptation that emerge from macro-level interactions. One exception is the research by Laube et al. [45] who found that international trade regimes and associated market competition imposed social limits on farmers adapting to water scarcity by taking up new agricultural technologies in Ghana.

Our study has several broad implications for adaptation policy and planning. First, by identifying important social limits with real consequences for adaptation outcomes, it highlights the need to consider ‘limits to adaptation’ in vulnerability assessments prior to implementation of particular strategies. Second, it shows that limits can emerge in social, economic and political domains that are different from the level at which people experience climate change and undergo adaptation (see also [46]). Hence, to overcome such limits requires engagement with a wide range of sectors and stakeholders. For instance, in the Great Barrier Reef case addressing relationships between reef and non-reef industries is important. Third, contrary to the literature’s relatively narrow focus on ecosystem-based adaptation to climate change our study indicates a general need to consider a broader suite of factors, which may include issues such as market processes, marketing, and improving representation and collective action.

Co-ordinated policy between stakeholders is also required to simultaneously address psycho-social and structural limits at multiple organisational levels. We hypothesised that at larger scales as values and institutions become more diverse, complex and contradictory limits would become more intractable [18]. Our analysis suggests, however, that the mutability of social limits from the perspective of stakeholders is more varied and depends on where stakeholders see opportunities for change. Reef stakeholders view limits arising from externalities emergent from land-use practices and underlying beliefs as particularly intractable. Whereas some existing market-based limits are expected to shift in response to effective local to global marketing campaigns.

The contextual nature of social limits to adaptation is important and highly challenging for management and policy. As articulated by Felgenhauer ([17]: 214) there are several options available to actors and policy makers upon reaching the social limits to adaptation including “investment in more of the same technology [approach], implementation of new and more effective adaptation, or transformational adaptation”. However, this implication that other options may be available to some if not all actors before transformation is required, creates a dilemma for assessing claims of residual, unavoidable loss and potential compensation (e.g., UNFCCC Warsaw International Mechanism). The IPCC fifth assessment report states that: “a limit is a point when an intolerable risk must be accepted; the objective itself must be relinquished; or some adaptive transformation must take place to avoid intolerable risk” ([8]: 906). Yet, the report also recognizes the significant ethical complexity of assessing what is an ‘intolerable risk’ across cultures.

There is, we argue, unresolved ambiguity in the science and policy of limits about what constitutes a ‘hard’ or ‘soft’ social limit and, by extension, what comprises unavoidable and unacceptable losses that necessitate transformation. What Klein et al. [8] may regard as ‘soft’ limits are currently experienced as effectively absolute limits in the Great Barrier Reef region given existing path dependencies and institutional inertia [18]. Yet, these limits apply to some but not all of the adaptation strategies available to particular industries. Reflecting our approach, Felgenhauer [17] puts forward the idea of an adaptation response ladder to depict how alternative strategies are employed as others reach their limit until no further options are available. He further suggests that once limits are passed failure may be linear or non-linear, and losses may be more or less recoverable. This interpretation of social limits contrasts the non-linear, threshold models advocated by, for example, Dow et al. [14,15]. We argue that understanding how limits differentially apply to sectors, strategies and particular climate impacts can help to prioritize adaptation investments and avoid strategies that are more prone to failure. Our approach also highlights, as above, that diverse interventions are required to overcome limits.

As well as revealing the specific nature of how limits to adaptation for reef-based industries unfold in the Great Barrier Reef region, our study uncovers different, more generalisable, mechanisms by which limits emerge. In our study, adaptation strategies failed to deliver desirable outcomes in three ways. First, reminiscent of economic limits, the effort (social costs) of pursuing a particular strategy exceeded the benefits received in terms of desirable outcomes. In the Coral Reef Fin Fish Fishery operators migrated north in the aftermath of an extreme weather event but for some businesses the benefits accrued did not mitigate the costs of catch declines. Second, the particular strategy did not actually address the real source of vulnerability. In the East Coast Trawl Fishery there is an assumption that vulnerability emerges from stock availability when it actually derives from market competition, hence effort buy-out strategies may fail. Third, the benefits derived from a particular strategy are undermined by external factors. Stewardship by reef industries can be significantly undermined by externalities from catchment land-use, which beyond a particular point are experienced as limits. These latter two pathways are not yet well recognised limits to adaptation and highlight the need to interrogate the diversity of social limits to adaptation.

## Supporting Information

S1 Table List of organisations invited to participate in this research. Respondents either attended a workshop in Townsville on 25^th^ March 2011 or Cairns on 01 April 2011, or were interviewed before the end of April 2011.
(DOCX)
