# Supplementary material for: Structural and Psycho-Social Limits to Climate Change Adaptation in the Great Barrier Reef Region
Source: PLoS One. 2016 Mar 9;11(3):e0150575. doi: 10.1371/journal.pone.0150575 (PMC4784939; doi:10.1371/journal.pone.0150575)
Supplement: S1 Table — Respondents either attended a workshop in Townsville on 25th March 2011 or Cairns on 01 April 2011, or were interviewed before the end of April 2011. (DOCX) [file pone.0150575.s001.docx]

| **Stakeholder organisation** | **Participated (Y/N)** |
| --- | --- |
| Agric-Science Queensland (DEEDI now DAFF) | Y |
| Cairns and Far North Environment Centre (CAFNEC) | Y |
| Cairns Marine | Y |
| Cairns Regional Council | Y |
| Commonwealth Scientific and Industrial Research Organisation (CSIRO) | Y |
| EcoFishers Queensland | Y |
| Fisheries Research Development Corporation (FRDC) | Y |
| Great Barrier Reef Marine Park Authority (GBRMPA) | N |
| Mackay Tourism | Y |
| North Queensland Dry Tropics (NQDT) | N |
| Ocean Watch | Y |
| Queensland Centre of Excellence for Climate Change (DERM now DEHP) | Y |
| Queensland Parks and Wildlife Service (DERM now DEHP) | Y |
| Fisheries Queensland (DEEDI now DAFF) | Y |
| Queensland Seafood Industry Association (QSIA) | Y |
| Queensland Tourism Industry Council (QTIC) | Y |
| Sunfish | Y |
| Terrain NRM | N |
| The Association of Marine Park Operators (AMPTO) | N |
| Traditional Owners | N |
| Tourism Queensland | Y |
| Tourism Whitsundays | N |
| Townsville City Council | N |
| WWF Australia | N |
